# Radiology residency across Europe: a fragmented yet converging path

**DOI:** 10.1186/s13244-026-02396-0

**Published:** 2026-09-25

**Authors:** Emanuele Neri, Salvatore Claudio Fanni, Dania Cioni

**Affiliations:** Department of Translational Research, Academic Radiology, University of Pisa, Pisa, Italy

**Keywords:** Curriculum, Education, Europe, Radiology, Teaching

## Abstract

**Abstract:**

Radiology has evolved into a central specialty at the core of modern medicine, with radiologists increasingly serving as integral members of multidisciplinary teams. Despite the specialty’s central role, the structure and content of radiology residency training remain highly variable across Europe. Differences persist in program duration, entry requirements, curriculum design, assessment modalities, and certification authorities, largely due to divergent national educational frameworks and healthcare policies. This fragmentation poses challenges to workforce mobility, training equivalence, and quality assurance. In response, the European Society of Radiology (ESR) has introduced harmonization initiatives, including the European Training Curriculum (ETC), the European Diploma in Radiology (EDiR), and the European Training Assessment Programme (ETAP), all aimed at promoting standardization while respecting national autonomy. This narrative review offers a comparative analysis of radiology training programs across European countries, with particular focus on admission pathways, curricular alignment with the ETC, implementation of research and subspecialty training, and variations in evaluation and certification. While most nations have adopted the ETC in whole or in part, practical implementation remains uneven. Entry mechanisms range from competitive national exams to local interviews, and while some programs include dedicated research time or thesis requirements, others lack structured scientific training. Furthermore, differences in legal and political frameworks influence how training is funded, organized, and certified. As Europe moves toward greater integration of specialist education, understanding these disparities is essential for developing a coherent, high-quality training landscape that ensures both clinical excellence and cross-border professional mobility.

**Key Points:**

- **Question** How heterogeneous are radiology residency structures and curricula across Europe, and to what extent do current frameworks address inconsistencies in training quality and standardization?
- **Findings** Radiology training across Europe remains markedly heterogeneous, while the European Training Curriculum, EDiR, and ETAP only partially mitigate variability in education, assessment, and quality standards.
- **Critical Relevance Statement** This narrative review critically evaluates persistent inconsistencies in European radiology training and demonstrates the limited but essential role of harmonization initiatives, supporting the need for stronger integration to ensure comparable competencies and improve clinical practice standards.

**Graphical Abstract:**

**Figure:**
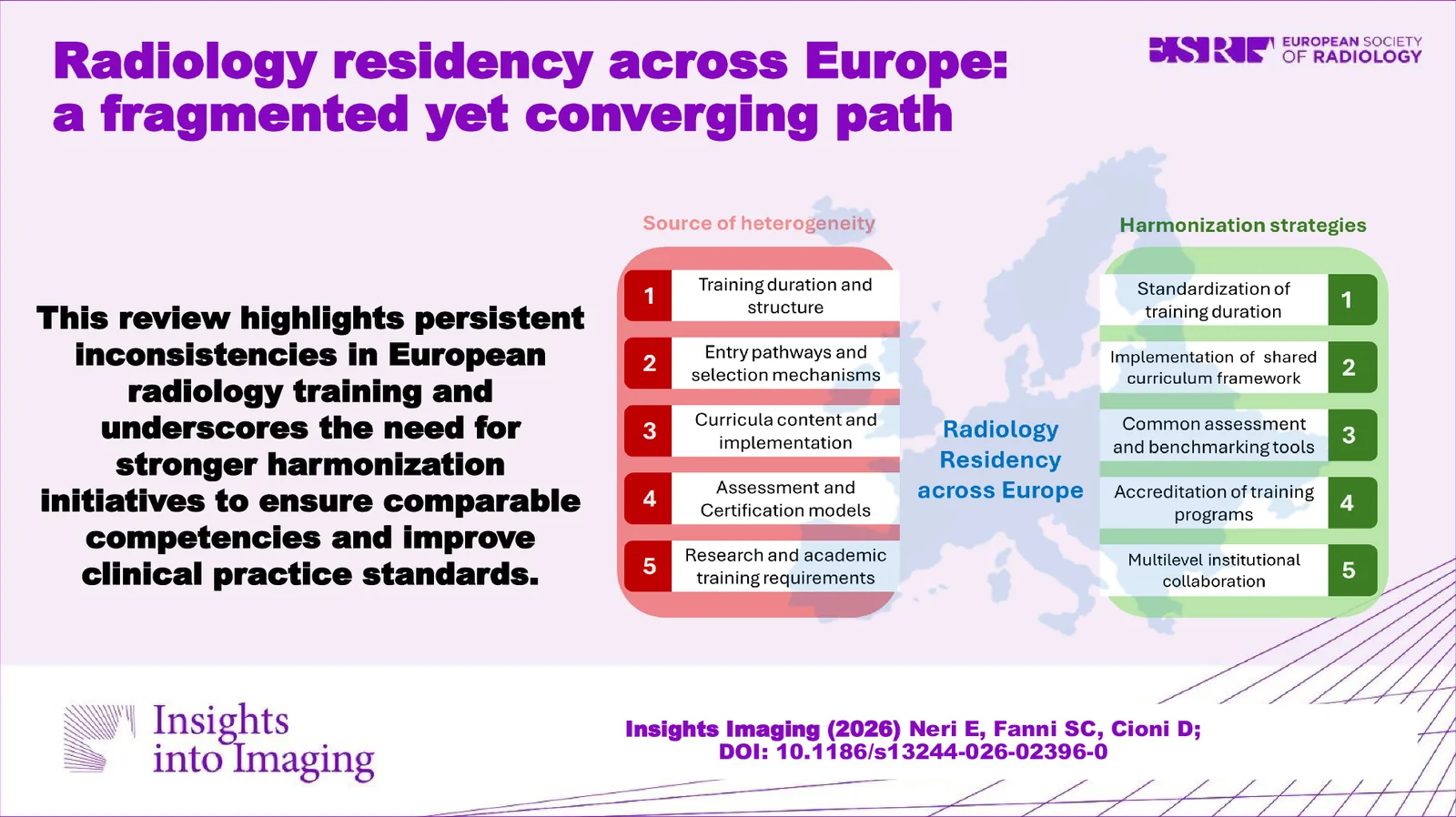

## Introduction

In recent years, radiology has emerged as a cornerstone of modern medicine, with the radiologist transitioning from a peripheral consultant role to a central figure in multidisciplinary clinical decision-making [1]. Radiologists now routinely engage in clinical decision-making, longitudinal care planning, and image-guided therapeutic interventions, reflecting their increasingly indispensable role in modern healthcare systems [1].

This shift in clinical relevance has placed heightened demands on postgraduate radiology training, requiring curricula to incorporate not only technical and interpretative expertise but also competencies in communication, leadership, ethics, and patient safety.

However, the pathway to meet all these requirements in Europe remains fragmented.

Although all EU Member States offer residency training in diagnostic and interventional radiology, national programs differ substantially in duration (ranging from 3 to 6 years), structure, entry requirements, content depth, and assessment methods. These differences reflect diverse legal frameworks, educational traditions, and health policy priorities, but they also contribute to persistent challenges in cross-border mobility, mutual recognition of qualifications, and quality assurance in specialist training.

The European Society of Radiology (ESR) has taken the lead in promoting harmonization through initiatives such as the European Training Curriculum (ETC), the European Diploma in Radiology (EDiR), and the European Training Assessment Programme (ETAP) [2, 3].

This educational review aims at critically examining the current state of radiology training across Europe, comparing structural models, identifying key divergences, evaluating the progress of integration strategies, and, last but not least, addressing the political and legislative context affecting postgraduate medical education.

## Duration and curriculum structure

The duration of radiology residency in Europe varies from 3.5 to 6 years, with a median of 5 years. Countries like Italy, Spain, and Bulgaria continue to offer 4-year programs, while others, such as Germany, France, the Netherlands, and the UK, adhere to 5-year frameworks [4, 5]. Some north European countries, including Sweden and Finland, have training paths extending to 5.5–6 years. The duration of training and other key characteristics of representative examples of the main organizational models of postgraduate medical education in Europe are summarized in Table 1.

**Table 1:** Key characteristics of representative European radiology training pathways, illustrating differences in training duration, admission systems, progression and assessment methods, and certification processes across selected countries

Country	Admission	Duration	Final assessment	Certification
Italy	National admission examination	4 years	Final thesis/discussion	University diploma
Austria	Local employment in accredited departments	6 years	Facharzt examination	Austrian Medical Chamber
Germany	Employment as Assistenzarzt	5 years	Chamber Facharzt examination	Regional Medical Chamber
United Kingdom	National recruitment (Oriel)	5 years (ST1–ST5)	ARCP + Royal College examinations	CCT and GMC Specialist Register
Spain	MIR national examination	4 years	Completion of residency portfolio	Ministry of Health
Belgium	University-based selection	5 years	University assessment	Federal recognition of specialist title
Switzerland	Direct application to accredited departments	5 years	SIWF/ISFM board examination	SIWF/ISFM diploma
France	National ranking (EDN)	5 years	DES completion and thesis	DES diploma and registration
Poland	LEK examination	5 years	PES specialty examination	Ministry of Health
Croatia	Public institutional recruitment	5 years	National final examination	Ministry of Health
Greece	Waiting-list system	5 years	National specialist examination	Ministry of Health

The European Commission-funded EU-REST (European Union Radiation, Education, Staffing & Training), under the Strategic Agenda for Medical Ionizing Radiation Applications (SAMIRA) [6] plan advocates a standardized 5-year residency to ensure adequate exposure to general and organ-based imaging, radiation safety, as well as emerging technologies [7]. Pre-residency internships are mandatory in several countries (e.g., Germany, Netherlands), where graduates must complete 1 year of general clinical practice before entering specialty training. Conversely, Italy and France allow direct transition from medical school to specialty training, integrating general medicine exposure within the early residency years [8].

This heterogeneity stems from differences in national minimum training requirements, with EU legislation currently recognizing a minimum of 4 years for specialty training in radiology under Directive 2005/36/EC [9] as the standard minimum duration, pending future revisions, and local decisions to modify training duration in order to better meet the growing demands of the discipline. While sufficient for formal professional qualification recognition, this standard lags behind clinical realities. The European Training Curriculum (ETC), endorsed by the ESR and the Union Europeenne des Medecins Specialistes (UEMS), outlines a comprehensive 5-year structure divided into three levels. Level I (years 1–3) includes broad-based general radiology training, covering all major imaging modalities and systems. Level II (years 4–5) emphasizes advanced interpretation, multidisciplinary integration, and the development of subspecialty competencies. Level III, even if not included within the ETC, offers fellowship-level curricula for organ/system-based specialties such as neuroradiology, interventional radiology, and musculoskeletal imaging [7]. The ESR European Training Curriculum is supported by 38 national radiology societies, although implementation as the formal basis of national training programmes is still not universal [7]. An ESR survey among training directors demonstrated broad awareness of the ETC and increasing interest in competency-based approaches, including entrustable professional activities, although implementation remains heterogeneous across Europe [2]. Indeed, curricular depth and implementation vary. For example, radioprotection and imaging physics are emphasized in German and Nordic programs with dedicated assessments, while others address them within broader clinical modules. In some countries, this topic is covered through dedicated courses lasting only a few weeks, whereas in others it is delivered through substantially more extensive modules. According to data collected by the ESR, radiation protection training during radiology residency can range from less than 2 total weeks to 16–24 total weeks, depending on the country [4]. This heterogeneity reflects the lack, to date, of a single unified standard, despite the existence of a European directive (Council Directive 2013/59/Euratom) obliging Member States to ensure adequate radiation protection education and training for all professionals involved. In practice, each country has implemented these requirements at a national level; the EU-funded ESR-coordinated project Medical Radiation Protection Education and Training (MEDRAPET) had already highlighted substantial differences in how European radiation protection regulations are applied within educational settings [10].

## Training providers and certification authorities

Radiology training in Europe is administered either through academic institutions or health authorities, depending on the national model. In university-based systems (e.g., Italy, Spain, France), residency is regulated by ministries of education and delivered within university hospitals. Trainees receive an academic degree (e.g., Diploma di Specializzazione in Italy or Diplôme d'Études Spécialisées in France). Conversely, in countries like the UK, Ireland, and Germany, training is hospital-centered, with oversight by professional colleges (e.g., Royal College of Radiologists) or medical chambers (Ärztekammern).

Selection into residency also varies across Europe [11]. In countries like Italy and Spain, competitive national entrance examinations, such as the Italian SSM (Scuola di specializzazione in medicina) and the Spanish MIR (Médico Interno Residente), are used to allocate residency positions across all medical specialties. Due to the high demand for radiology, only top-ranked candidates are typically able to secure placements in this field. Other countries rely on local applications, interviews, or combined systems. Funding mechanisms differ as well: some trainees are salaried employees (e.g., UK, Germany), while others receive stipends or academic scholarships (e.g., some Eastern European states).

Assessment during training ranges from continuous performance evaluations, case logbooks, and interim oral or written tests to formal national exit exams. In the United Kingdom, assessment is largely based on the Fellowship of the Royal College of Radiologists (FRCR) examinations. The First FRCR examination consists of separate anatomy and physics modules, while the Final FRCR includes written examinations (Part 2 A) and a Part 2B assessment comprising rapid reporting, long-case reporting, and oral examinations [12]. In Germany, a single final oral exam with regional chamber examiners is required. In Italy, residents must defend a research-based thesis in a public academic exam. However, some countries do not require a formal final exam, instead relying on accumulated assessments and mentor evaluations (Table 1) [13].

## Research, subspecialization, and fellowships

Scientific training is increasingly regarded as an essential component of radiology education. Around 40% of European countries formally require residents to engage in research, with activities ranging from clinical case reports to the completion of a thesis or publication in peer-reviewed journals. Countries such as Germany, Switzerland, and the Netherlands integrate protected research time into the residency curriculum, while in Italy and France, a final thesis is mandated as part of the academic qualification process. Nevertheless, recent data from a survey published by the ESR Radiology Trainee Forum highlight persistent gaps in research exposure during training [14]. In a survey of 159 trainees from 29 countries, only 7.5% held a PhD at the time of participation, and nearly half (47.8%) had never authored a PubMed-indexed publication. Furthermore, a substantial proportion reported limited formal instruction in core research skills: 79% had not received dedicated training in statistics, 34.6% had never been taught how to critically appraise a scientific paper, and 36.5% had not been encouraged to engage in research activities during residency.

Regarding subspecialization, despite the rise of organ-based medicine, the recognition and structuring of radiology subspecialties remain inconsistent. As of 2023, 25 countries officially recognize at least one radiology subspecialty, but the scope varies widely, from only interventional radiology in Greece to 10–12 specialties in Slovenia, Switzerland, and Finland. Frequently recognized fields include neuroradiology, interventional radiology, pediatric radiology, and breast imaging.

In most cases, subspecialty recognition requires additional fellowship training (1–2 years), often without formalized national accreditation. Some countries provide subspecialty diplomas via professional societies (e.g., France’s advanced interventional radiology degree). Elsewhere, these remain informal job market distinctions. The ESR has developed Level III curricula and supports European-wide diplomas for various specialties through affiliated societies (e.g., EBIR, ESUR, ESGAR).

A 2023 ESR survey indicated that nine of twelve countries currently lacking subspecialty diplomas expressed interest in future implementation. However, policy and funding barriers remain significant. Without centralized subspecialty certification, radiologists often rely on clinical experience, peer reputation, or international certifications to demonstrate expertise.

## Harmonization through ESR initiatives

The EDiR is the flagship initiative of the ESR for certification harmonization [3]. Administered by the European Board of Radiology, it tests knowledge and practical skills aligned with the ETC [15]. As of 2023, EDiR is accepted as equivalent to national board exams in Poland and Turkey, and as a partial replacement in Finland, the Netherlands, and Croatia. The ESR recommends its adoption as a standard exit examination.

Other initiatives include the above-mentioned ETAP, which evaluates training centers against ESR quality benchmarks, and the Accreditation Council in Imaging (ACI), which harmonizes continuing education standards (https://doi.org/10.1186/s13244-025-02054-x). Subspecialty diplomas in neuroradiology, head and neck, breast imaging, musculoskeletal, gastrointestinal, and interventional radiology are also expanding under ESR guidance [16]. Radiology training in Europe remains a heterogeneous field shaped by diverse educational traditions, legislative frameworks, and healthcare structures. However, the trajectory toward convergence is clear. A unified 5-year curriculum, standardized certification via the EDiR, and recognized pathways for subspecialty development are attainable goals.

Beyond educational considerations, harmonization of radiology training also has important implications for professional mobility within the European Union. Comparable curricula, assessment standards, and competency frameworks may facilitate the recognition of qualifications and support workforce mobility across member states. However, curricular convergence alone may not fully overcome existing barriers. National licensing procedures, language requirements, professional registration systems, and country-specific recruitment mechanisms continue to influence access to specialist positions. Consequently, educational harmonization should be considered a necessary but not sufficient condition for achieving unrestricted mobility of radiologists within Europe.

**Fig. 1:**
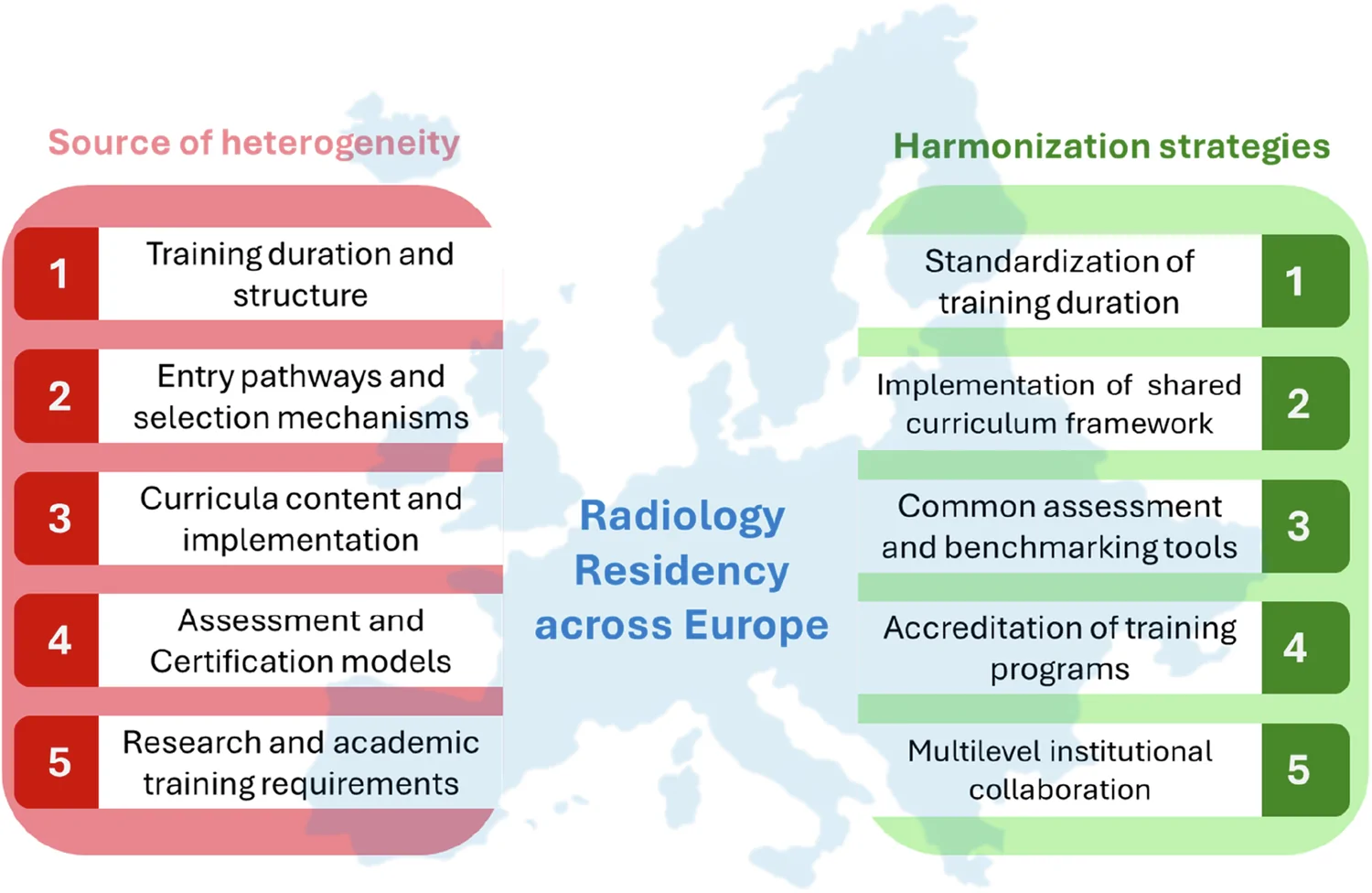
Current source of heterogeneity and harmonization strategies in radiology residency across Europe

Achieving effective harmonization of radiology training across Europe will require several key steps. These include the revision of EU legislation to formalize a 5-year minimum duration for specialty training; the widespread implementation of the ETC and endorsement of the EDiR as a standard of reference; and the structured recognition of radiological subspecialties within national and European regulatory frameworks (Fig. 1).

An additional area deserving future discussion concerns, beyond EDiR, the role of European subspecialty diplomas in the accreditation of subspecialized radiologists. Although these examinations currently serve as voluntary certifications, they provide standardized competency assessments that may help reduce heterogeneity in subspecialty training across Europe. At present, making such examinations mandatory for subspecialty practice would be difficult because of the considerable differences in national legislation, healthcare organization, and specialist recognition systems. Nevertheless, European subspecialty diplomas could progressively evolve into recognized quality benchmarks for specific positions, particularly in highly specialized fields such as neuroradiology, interventional radiology, or breast imaging. A gradual implementation strategy, based on voluntary adoption, national recognition, and eventual integration into fellowship or post-residency training pathways, may represent a realistic approach toward greater harmonization.

Harmonization efforts should also encompass enhanced investment in training infrastructure, particularly protected time for research and comprehensive radioprotection education. Crucially, sustained collaboration among ministries, universities, accreditation bodies, and professional societies will be indispensable to align standards while accommodating the diversity of national systems. ESR’s leadership is vital to sustaining this momentum. As radiology continues to evolve with artificial intelligence, hybrid imaging, and molecular diagnostics, robust and harmonized training will be essential to ensure ESR’s diagnostic excellence and patient safety across Europe.

## Abbreviations

EDiR: European Diploma in Radiology
ESR: European Society of Radiology
ETAP: European Training Assessment Programme
ETC: European Training Curriculum
SAMIRA: Strategic Agenda for Medical Ionizing Radiation Applications

## References

[1] Brady AP, Beets-Tan RG, Brkljačić B, Catalano C, Rockall A, Fuchsjäger M (2022) The role of radiologist in the changing world of healthcare: a White Paper of the European Society of Radiology (ESR). Insights Imaging 13:100. 10.1186/s13244-022-01241-4 PMC9167391 35662384

[2] Hirvonen J, Becker M, Aronen HJ (2023) Resident education in radiology in Europe including entrustable professional activities: results of an ESR survey. Insights Imaging 14:139. 10.1186/s13244-023-01489-4 PMC10444922 37606861

[3] European Board of Radiology (EBR) (2018) The European Diploma in Radiology (EDiR): investing in the future of the new generations of radiologists. Insights Imaging 9:905–909. 10.1007/s13244-018-0665-7 PMC6269329 30291525

[4] Brady AP, Paulo G, Brkljacic B, Loewe C, Szucsich M, Hierath M (2025) Current status of radiologist staffing, education and training in the 27 EU Member States. Insights Imaging 16:59. 10.1186/s13244-025-01925-7 PMC11910488 40088348

[5] Rehani B, Zhang YC, Rehani MM et al (2017) Radiology education in Europe: analysis of results from 22 European countries. World J Radiol 9:55. 10.4329/wjr.v9.i2.55 PMC5334502 28298965

[6] European Commission (2021) Available via https://circabc.europa.eu/ui/group/8f5f9424-a7ef-4dbf-b914-1af1d12ff5d2/library/656af0cf-46ef-4b28-a9b7-1117e73ab19f/details. Accessed 21 Jan 2026

[7] Brady AP, Loewe C, Brkljacic B, Paulo G, Szucsich M, Hierath M (2025) Guidelines and recommendations for radiologist staffing, education and training. Insights Imaging 16:57. 10.1186/s13244-025-01926-6 PMC11904080 40074928

[8] Sendra-Portero F, Souto M, Becker M, Goh V (2023) Undergraduate radiology education in Europe in 2022: a survey from the European Society of Radiology (ESR). Insights Imaging 14:37. 10.1186/s13244-023-01388-8 PMC9958208 36826721

[9] The European Parliament and the Council of the European Union (2005) Available via https://eur-lex.europa.eu/eli/dir/2005/36/oj/eng. Accessed 21 Jan 2026

[10] Available via https://www.eurosafeimaging.org/medrapet. Accessed 21 Jan 2026

[11] Hagelsteen K, Pedersen H, Bergenfelz A, Mathieu C (2021) Different approaches to selection of surgical trainees in the European Union. BMC Med Educ 21:363. 10.1186/s12909-021-02779-5 PMC8243060 34193137

[12] Royal College of Radiologists. Clinical radiology examinations. Available via https://www.rcr.ac.uk/exams-training/rcr-exams/clinical-radiology-exams/. Accessed June 2026

[13] European Society of Radiology (2006) Radiological training programmes in Europe. Available via https://www.myesr.org/app/uploads/2023/08/ESR_brochure_05.pdf. Accessed June 2026

[14] Klontzas ME, Reim M, Afat S, Podzniakova V, Snoeckx A, Becker M (2024) Research training during radiology residency: findings from the ESR Radiology Trainee Forum survey. Insights Imaging 15:236. 10.1186/s13244-024-01812-7 PMC11458849 39373762

[15] Ertl-Wagner B (2014) Europäisches curriculum für die weiterbildung in der radiologie. Radiologe 54:1106–1110. 10.1007/s00117-014-2685-8 25398573

[16] Rupreht M, Ricci P, Prosch H, Adriaensen MEAPM (2023) Subspecialisation in radiology in Europe, a survey of the accreditation council of imaging. Insights Imaging 14:159. 10.1186/s13244-023-01481-y PMC10519886 37749296

